# Rapidly Progressive Mucormycosis Presenting as Giant Cell Arteritis as a First Presentation of Diabetes in a Woman With Diabetic Ketoacidosis: A Case Report and Review of the Literature

**DOI:** 10.1155/crdi/4066148

**Published:** 2026-05-13

**Authors:** Suha Nori, Dua Nori, Hala O. Abdallah, Ansam Nafah, Muath N. M. Salman, Haitham Hamadneh, Reem Shihab

**Affiliations:** ^1^ Department of Medicine, Faculty of Medicine and Health Sciences, An-Najah National University, Nablus, State of Palestine, najah.edu; ^2^ Department of Radiology, Istishari Arab Hospital, Ramallah, State of Palestine; ^3^ Department of Radiology, Nablus Specialty Hospital, Nablus, State of Palestine

**Keywords:** case report, diabetic ketoacidosis, giant cell arteritis mimic, intracerebral hemorrhage, mucormycosis

## Abstract

**Background:**

Disseminated mucormycosis is a rare, angioinvasive fungal infection predominantly affecting immunocompromised individuals, especially those with poorly controlled diabetes.

**Case Presentation:**

A 57‐year‐old Palestinian woman presented with sudden unilateral blindness, ophthalmoplegia, facial numbness, and ptosis, with hyperglycemia. Early brain MRI/MRA/MRV findings were normal, suggesting that giant cell arteritis, or other autoimmune vasculitis, was suspected; thus, IV steroids were administered. Subsequently, her condition progressed to bilateral fixed pupils, complete ophthalmoplegia, and palatal black necrosis. Subsequent imaging and sinus biopsy confirmed disseminated mucormycosis. Treatment included intravenous Amphotericin B, anticoagulation, functional endoscopic sinus surgery, and later left eye enucleation. Despite maximal therapy, she developed a large hemorrhagic venous infarction and ultimately died.

**Conclusions:**

Mucormycosis can initially mimic autoimmune or vascular neuro‐ophthalmic disease and may present with normal imaging. In acute vision loss with hyperglycemia, maintaining early suspicion and avoiding corticosteroids until fungal infection is excluded are essential to prevent rapid, fatal progression.

## 1. Background

Mucormycosis is a rare, opportunistic angioinvasive fungal infection caused by members of the Mucorales order. It is recognized as the third most common angioinvasive fungal disease, following candidiasis and aspergillosis [1]. It predominantly affects immunocompromised individuals, particularly those with uncontrolled diabetes, and most commonly presents as rhino‐orbito‐cerebral mucormycosis (ROCM) disease [2]. The pathogenesis is marked by vascular invasion, thrombosis, ischemia, and tissue necrosis [1], which may extend to involve the central nervous system and other organs [2]. Mucor exhibits a strong predilection for blood vessels, especially arteries, and its invasion of vessel walls is a hallmark feature of the infection [2]. Disseminated mucormycosis, though rare, carries a near‐100% mortality rate, particularly when multiple organs are involved [3]. It usually occurs in neutropenic patients with hematologic malignancies, transplants, or deferoxamine therapy, and poorly controlled diabetes [3]. Histopathological examination remains the diagnostic gold standard [3]. Diagnosis relies on identifying the organism, which appears as broad, aseptate hyphae with right‐angle branching [4]. Management requires aggressive therapy, including antifungal treatment with Amphotericin B and surgical debridement or resection of affected tissues or organs [4].

Here, we present a case of disseminated mucormycosis in a previously undiagnosed diabetic patient. Unlike typical presentations, the initial MRI, MRA, and MRV were normal, delaying the diagnosis, while early clinical features mimicked giant cell arteritis. The patient developed simultaneous involvement of the optic nerves, multiple cranial nerves, and the splenic vein, culminating in bilateral irreversible blindness, intracerebral hemorrhage, and widespread vascular complications. Additionally, empirical corticosteroid therapy likely accelerated fungal dissemination, highlighting a critical diagnostic pitfall. Collectively, these features illustrate an atypical and fulminant course that has rarely been reported, providing important insights into early recognition and management of disseminated mucormycosis.

## 2. Case Presentation

A 57‐year‐old Palestinian female with no known prior medical history presented with rapidly progressive unilateral visual loss. Her initial symptoms, all developing within 24 h, included left‐sided facial numbness, complete vision loss in the left eye, and ophthalmoplegia. She sought evaluations at multiple healthcare facilities before presenting to our center.

She had no known family history of autoimmune or vascular diseases. Psychosocial history was unremarkable. She was not on chronic medications and had no prior diagnosis of diabetes. No previous major interventions were reported.

Initial examination demonstrated complete vision loss in the left eye with ptosis, a fixed mid‐dilated pupil, minimal conjunctival injection, severely restricted extraocular movements, a pale optic disc on fundoscopy, and left mouth deviation with upper facial sparing. The oral cavity showed no palatal discoloration. The patient was afebrile on presentation with no signs of meningismus.

The patient had no known history of diabetes; however, her laboratory testing revealed marked hyperglycemia with an RBS of 310 mg/dL and newly diagnosed diabetes with an HbA1c of 14.1%, along with diabetic ketoacidosis (DKA) characterized by ketonuria (+3) and metabolic acidosis (pH 7.32, HCO_3_^-^ 10.3 mmol/L). Inflammatory markers were elevated, including a CRP of 162 mg/L and neutrophilia at 85.5%. Additional abnormalities included mild hyponatremia (Na^+^ 132 mmol/L), hypokalemia (K^+^ 3.27 mmol/L), and thrombocytopenia (platelets 104 × 10^9^/L). The autoimmune panel showed a positive ANA (1:80) by indirect immunofluorescence; the extractable nuclear antigen (ENA) panel was negative, with negative anti‐dsDNA and anti‐Sm, and a negative thrombophilia screen.

Metabolic management included electrolyte correction for severe hypokalemia (K^+^ 2.6 mmol/L) and treatment of DKA with insulin therapy consisting of 15 units of rapid‐acting insulin three times daily and 12 units of basal insulin daily.

**FIGURE 1:**
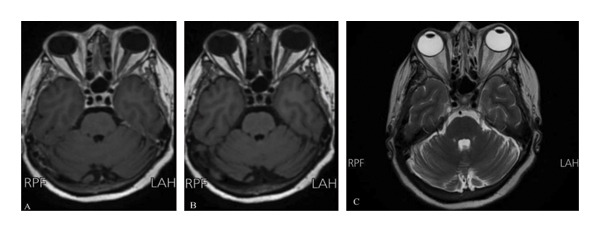
Brain MRI: T1‐weighted postcontrast (A), T1‐weighted precontrast (B), and T2‐weighted (C) images demonstrate no intracranial abnormalities or enhancing space‐occupying lesions.

**FIGURE 2:**
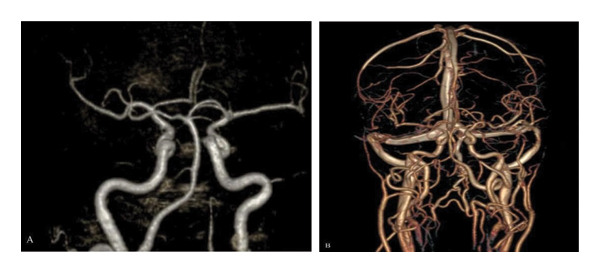
Brain angiography demonstrates no vascular or structural abnormalities, in both MRA (A) and MRV (B).

MRI (Figure 1), MRA, and MRV (Figure 2) were normal. Thus, high clinical suspicion of GCA was made, and empirical methylprednisolone (1 g IV 1 × 1 for 3 days) and ceftriaxone (1 g IV 1 × 2) were initiated. However, no clinical improvement was observed.

**FIGURE 3:**
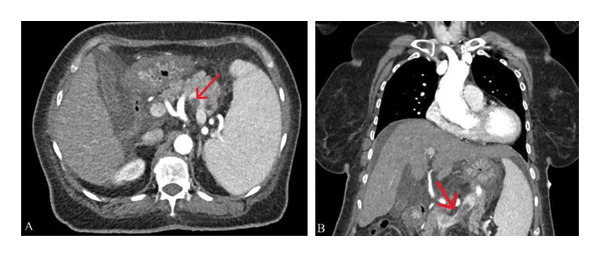
Axial (A) and coronal (B) contrast‐enhanced chest CT scans showing filling defects within the splenic vein.

On Day 3, she was transferred to the neuro ICU with bilateral fixed pupils, complete ophthalmoplegia, and new palatal black discoloration. Splenic vein thrombosis was detected on abdominal CT scan (Figure 3). Anticoagulation with enoxaparin (80 mg subcutaneously, 2 times daily for 2 weeks) was initiated. Clinical suspicion of mucormycosis was considered. Amphotericin B deoxycholate (50 mg intravenously 1 time daily for 1 month) and Fungizone 50 mg diluted in 500 mL of 5% dextrose over 6 h, which switched after 3 days to posaconazole, starting with a loading dose of 300 mg twice daily on Day 1, followed by 300 mg once daily, planned for at least 6 months, were initiated.

Histopathological examination of functional endoscopic sinus surgery (FESS) biopsies revealed nonseptate, broad hyphae at an angle of 90, confirming mucormycosis. Unfortunately, molecular diagnostic testing such as PCR for Mucorales was not available in our hospital at the time of diagnosis. Therefore, the diagnosis was based on the histopathological examination showing positive PAS, combined with clinical and radiological findings. Lumbar puncture (LP) was deferred due to thrombocytopenia.

**FIGURE 4:**
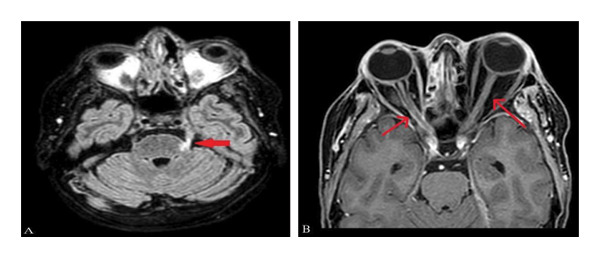
Brain MRI showing the findings suggestive of infectious neuritis involving the intracranial portion of the left trigeminal nerve (A) and bilateral optic nerves (B).

**FIGURE 5:**
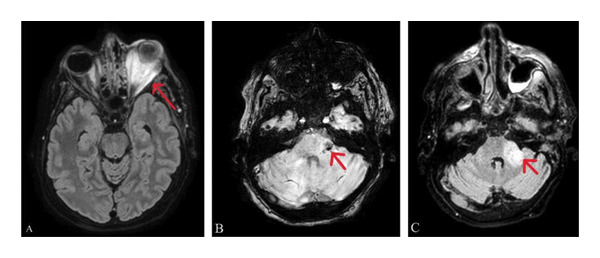
(A) Axial FLAIR image showing severe left orbital inflammation with panophthalmitis. (B) Axial SWI and (C) FLAIR images showing pontine and left middle cerebellar peduncle involvement with hemorrhagic foci.

**FIGURE 6:**
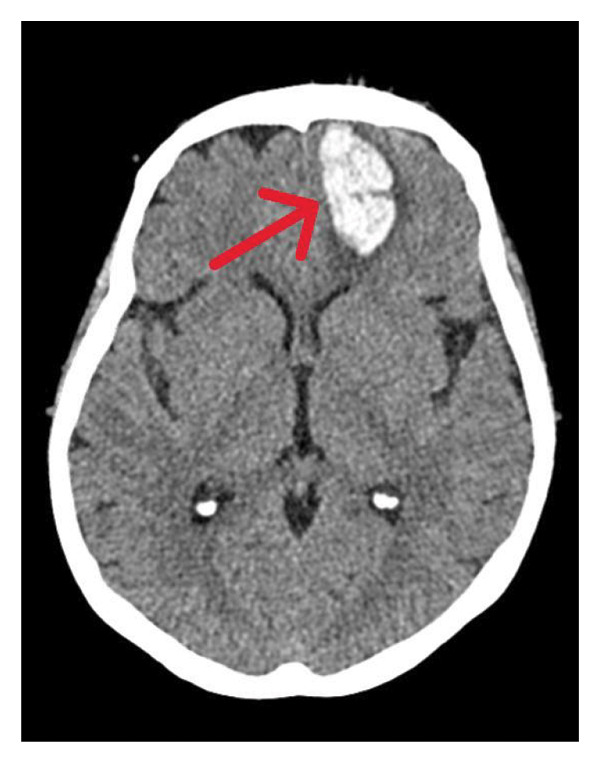
Noncontrast brain CT showing a large left frontobasal intracerebral hemorrhage, measuring approximately 2 × 3.8 × 4.7 cm, with no midline shift or significant ventricular compression.

Despite aggressive medical therapy, the disease progressed, with worsening orbital cellulitis with no light perception bilaterally. In the right eye, there was a flat retina and a pale, atrophic optic nerve. In the left eye, the cornea was opaque, obscuring the posterior segment, indicating bilateral irreversible blindness. MRI (Figure 4) showed bilateral optic and trigeminal nerve involvement, and brain CT (Figure 5) showed progressive orbital and CNS extension (pons, cerebellar peduncle, and cerebellum) with pansinusitis and mastoiditis. She continued to deteriorate with fever, facial swelling, and odynophagia; thus, left eye enucleation on Hospital Day 14 was performed. One day following enucleation, the patient developed seizures secondary to left frontobasal intracerebral hemorrhage (Figure 6), likely due to venous infarction. Management included initiation of levetiracetam for seizure control and mannitol for intracranial pressure management, and enoxaparin was stopped. During the later course of hospitalization, sputum culture grew extended‐spectrum beta‐lactamase (ESBL) producing *Klebsiella pneumoniae*, and antimicrobial therapy was escalated to meropenem 1 g intravenously every 8 h. By Day 25, the patient developed progressive hemodynamic instability and died despite maximal supportive measures.

**FIGURE 7:**
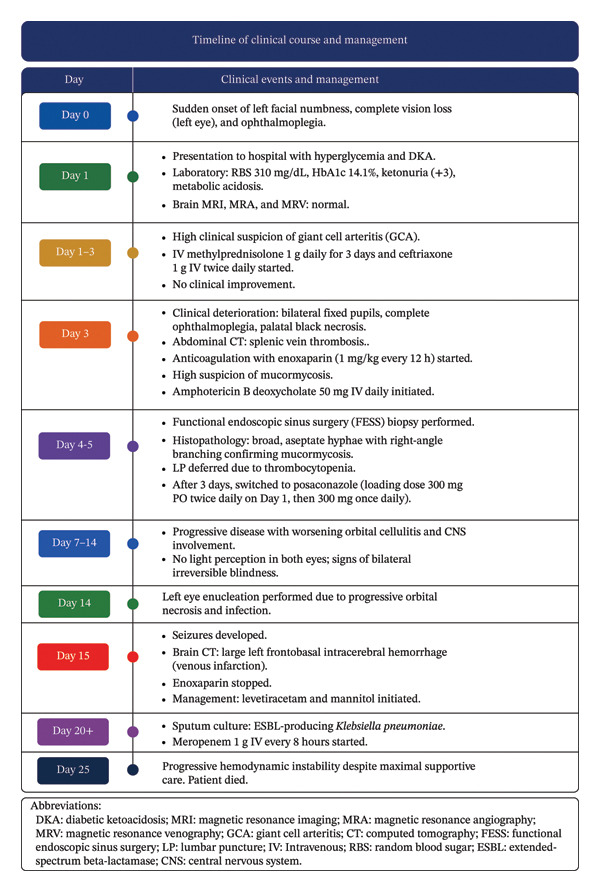
Timeline of the patient’s clinical course, investigations, management, and outcomes.

The sequence of events from presentation to outcome is illustrated in Figure 7.

## 3. Discussion and Conclusions

Mucormycosis’s ischemic symptoms and arterial invasion can make it clinically similar to vasculitides such as GCA. The course of the infection is greatly accelerated by this overlap, which can result in an inaccurate diagnosis and inadvertent corticosteroid treatment. Corticosteroids, which also promote hyperglycemia, impede neutrophil chemotaxis and phagocytosis, and decrease host defense, enable fungal growth and vascular penetration. Two examples that showed this relationship were reported in the literature, including a 75‐year‐old man with Type 2 diabetes mellitus who experienced severe headaches and complete eyesight loss. High‐dose corticosteroids were initially used to treat his suspected GCA, but his symptoms rapidly deteriorated, and a biopsy revealed mucormycosis. The disease progressed to cerebral involvement despite amphotericin treatment, demonstrating how corticosteroids hasten tissue necrosis [5].

The initial clinical impression was GCA, given acute, rapidly progressive unilateral vision loss, ophthalmoplegia, and associated cranial nerve findings. Brain MRI, MRA, and MRV were performed to rule out other differential diagnoses and were unremarkable. In addition, typical early signs of invasive mucormycosis, such as palatal necrosis, were not present at the time of presentation, which reduced the initial suspicion of an invasive infectious process. Based on this provisional diagnosis of GCA, empirical high‐dose methylprednisolone was initiated. The article acknowledges this as a diagnostic pitfall, particularly in the context of newly diagnosed DM with DKS during the time of current hospitalization, where an underlying infection should be carefully excluded. However, the early clinical features closely mimicked GCA, which led to the initiation of steroid therapy before mucormycosis was identified. We further note that corticosteroid administration may have accelerated the progression of the fungal infection.

Two additional cases highlight the harmful influence of immunosuppression on the disease progression. One described a 47‐year‐old woman with iatrogenic Cushing syndrome who was on continuous steroids and developed ROCM with multiple cranial‐nerve palsies [6]. Another unexpected case involved disseminated mucormycosis following pacemaker placement and a brief course of oral corticosteroids; the patient passed away less than a week after the definitive diagnosis [7].

These results highlight the importance of caution when administering steroids to diabetic or immunocompromised individuals who have acute ocular or ischemic symptoms, as was the case in our instance.

ROCM can cause orbital apex syndrome (OAS), which is characterized by proptosis, ophthalmoplegia, and vision impairment due to the involvement of Cranial Nerves II, III, IV, VI, and the maxillary division of V [8]. It was observed in our patient, exhibiting more extensive involvement than that seen in OAS, including Cranial Nerve VII. Facial nerve involvement in mucormycosis is rarely described, as most cases primarily involve Cranial Nerves II, III, IV, V, and VI in OAS. A similar rare presentation was reported by Bhatt et al. [6], who described a 47‐year‐old woman with periorbital pain, ptosis, and upper‐facial palsy due to mucormycosis. The presence of upper‐facial palsy in our patient, therefore, represents an unusual neurological manifestation that may reflect perineural spread of the fungus, a mechanism that has been infrequently documented.

The infection’s angioinvasive nature can also cause vascular occlusion and intracerebral or subarachnoid hemorrhage, as reported by Munoz et al. [9] Rhizopus oryzae–induced vascular invasion caused a 48‐year‐old man with DKA to exhibit abrupt hemiparesis and cerebral bleeding. These examples highlight the pathogen’s dual neurological and vascular tropism, as demonstrated by our patient, who had upper‐facial palsy and cerebral bleeding.

The aggressive course of disseminated disease was highlighted in a case report published in The Pediatric Infectious Disease Journal describing about a 16‐year‐old girl with DKA who developed disseminated mucormycosis that affected her lungs, brain, and spleen. She was treated with Amphotericin B and surgical debridement, but her prognosis was poor [10].

Another instance of chronic disseminated mucormycosis was documented by Nolan et al. [11] in a male diabetic patient aged 61 who also had epistaxis, lung infiltrates, and renal failure. After worsening, the patient died after receiving a misdiagnosis of Wegener granulomatosis; a biopsy showed mucormycosis, and an autopsy showed extensive ischemic necrosis of the pleura, lungs, kidneys, and blood vessels. While the lungs and brain are most frequently affected, splenic involvement is exceedingly uncommon [4].

In our case, diagnostic challenges included a normal initial MRI/MRA/MRV that obscured early invasive fungal spread, early clinical features that mimicked GCA leading to empirical steroid use that accelerated the fungal progression, thrombocytopenia that limited the ability to perform LP, and the delayed appearance of palatal necrosis, which is an important clinical clue for mucormycosis, that emerged only on Day 3.

While several reports have described ROCM presenting with either cranial neuropathies or vascular complications, to our knowledge, the coexistence of upper‐facial palsy, perineural spread, splenic vein thrombosis, and hemorrhagic venous infarction in a single patient has not been reported before. This constellation of findings expands the known neurological and vascular spectrum of disseminated mucormycosis. Early recognition is challenging due to nonspecific initial symptoms, particularly in patients with an unknown history of diabetes, but it is critical for improving outcomes. The case highlights the need for extreme caution when initiating corticosteroid therapy in diabetic or immunocompromised patients presenting with acute ocular or ischemic symptoms before infectious causes have been excluded.

**Nomenclature**
LP: Lumbar puncture
DKA: Diabetic ketoacidosis
FESS: Functional endoscopic sinus surgery
OAS: Orbital apex syndrome
GCA: Giant cell arteritis
ROCM: Rhino‐orbito‐cerebral mucormycosis
ENA: Extractable nuclear antigen
ESBL: Extended‐spectrum beta‐lactamase

## Author Contributions

Reem Shihab: introduction.

Hala O. Abdallah: case presentation.

Hala O. Abdallah, Dua Nori, and Reem Shihab: data collection.

Haitham Hamadneh and Muath N. M. Salman provided the radiological images and their corresponding reports.

Dua Nori, Reem Shihab, Suha Nori, and Ansam Nafah: literature review and discussion.

## Funding

The authors received no funding for this case report.

## Disclosure

All authors read and approved the final manuscript.

## Ethics Statement

Informed consent was obtained from the patient’s family included in the study.

## Consent

Written informed consent was obtained from the patient’s family for publication of this case report and any accompanying images. A copy of the written consent is available for review by the Editor‐in‐Chief of this journal.

## Conflicts of Interest

The authors declare no conflicts of interests

## Data Availability

The data that support the findings of this study are available from the corresponding author upon reasonable request.
